# How pom cheerleading improves the executive function of preschool children: the mediating role of speed and agility

**DOI:** 10.1186/s40359-022-00944-z

**Published:** 2022-10-19

**Authors:** Heng Wang, Wanying Ge, Chenyang Zhu, Yafang Sun, Shuguang Wei

**Affiliations:** 1 College of Physical Education, Henan Normal University, Xinxiang, 453007 China; 2 Department of Psychology, College of Education, Hebei Normal University, Shijiazhuang, 050024 China

**Keywords:** Pom cheerleading, Preschool children, Executive function, Speed, Agility

## Abstract

Physical exercises can improve individuals’ physical health and cognition, but the internal influence path is unclear. This study aims to examine the influence of pom cheerleading training on physical fitness and executive function of preschool children and explore the relationship between sports training, physical fitness, and executive function. We selected seventy-one preschool children and divided them into the experimental group (n = 36) and the control group (n = 35). The experimental group kept a 12-week pom cheerleading training, and the exercises of the control group remained normal. Children’s physical fitness and executive function were tested, in one week before and after the experiment, respectively. Results of repeated measurements analysis of variance and structural equation model test showed: (1) after 12-week pom cheerleading training, in terms of physical fitness, the experimental group has a significant improvement over the control group on agility and speed; in terms of executive function, the inhibitory control and working memory of the experimental group were significantly enhanced over the control group. (2) Speed quality plays a partial mediating role between pom cheerleading training and inhibitory control; agility plays a major mediating role between pom cheerleading training and working memory. It is concluded that physical exercise can directly improve preschool children’s executive function, and indirectly enhance executive function mediated by physical fitness. Furthermore, structured and systematic physical education should be adopted for preschool children to cultivate their interest in sports and enhance their cognition.

## Introduction

In the Global Action Plan on Physical Activity 2018–2030 issued by World Health Organization [1], it is pointed out that if people want to improve their current and future health, they should keep exercising. It highlighted the health-promoting effect of physical exercise.

However, the lifestyle and living philosophy have dramatically changed as the information age develops fast, especially with the widespread visualized e-devices and their penetration into lower-aged children. Additionally, the traditional sports teaching is known as a “fragmented” sports teaching, which cannot cultivate preschool children’s interest in sports learning and physical exercise habits, resulting in less participation in sports, and prolonged sitting time among preschool children, which will severely affect their physical health and cognitive ability. The specific embodiment includes: a decrease in physical fitness, increase in myopia rate, overweight or obesity, and the younger age trend of adult diseases [2–4]; in terms of the cognitive ability, distracted attention, worse control ability or hyperactivity [5, 6]. To change this situation, we should enhance children’s physical exercise, and improve their physical and mental health through systematic motor skill training.

Many researchers have confirmed that physical exercise can improve the physical fitness of children [7, 8]. Sports intervention for different individuals, such as healthy children [9] or children with obesity [10], developmental retardation [11], or hearing impairment [12], can exert a positive effect on their physical fitness.

According to the theory of Information Processing [13], physical exercise or training can make children focus on the information processing when they complete certain movements to improve the performance of motor learning and control, and then enhance children’s cognition.

Executive function (EF), as the core element of cognition, is mainly affected by the prefrontal cortex. It is a high-level cognitive function that enables individuals to exclude or filter irrelevant information and to lead purposeful and orderly behaviors [14]. It is also central to the emotional, cognitive and social functions, and serves as a critical component of intellectual activity, reasoning, problem-solving, and learning ability. Executive function includes three cognitive components: inhibitory control, working memory, and cognitive flexibility [15–17].

Research on the brain has confirmed that physical exercise can improve the function of the prefrontal cortex [18]. Furthermore, some studies have found that short-term one-time or long-term physical exercises can improve children’s executive function, especially low- and moderate-intensity exercises containing cognitive activities [19, 20]. Meanwhile, the intervention of specific exercise projects, such as ball games [21, 22], swimming [23], yoga [24], or sports games [25, 26], can all improve children’s executive function, especially for children with worse executive function. Meta-analysis [27] also found that physical exercise intervention can significantly affect children’s cognitive ability, mainly reflected in executive function. Researchers suggested that changes in brain structure and function through physical exercise affect cognitive function [28], such as increasing the volume of the gray matter in the frontal lobe and hippocampus [29], reducing gray matter injury [30], increasing the volume of the cerebral cortex and basal ganglia [31]. Batouli [32] believes that there may be two mechanisms to improve the cognitive ability: one is that physical exercise participates in the neural circuit of cognitive function [29]; the other is that physical exercise increases “cerebrovascular reserve” [33].

Furthermore, some studies have revealed that physical fitness is closely related to the brain maturity and cognitive development. According to the theory of Information Processing [13], in the early stage of learning, the brain processes information directly based on stimulus or the sensory input from the outside. This processing method is mainly from bottom to top. Motor skills are expressed through physical fitness, and the performance is to some extent depending on how accurately brain controls muscle. Therefore, researchers suggest that physical fitness is significantly associated with performance on certain cognitive tasks [34].

For example, it was found that there is a negative correlation between body weight and academic performance [35]. Studies on overweight and obese children discovered that agility was positively correlated with cognitive flexibility and inhibitory control, muscle strength was associated with planning ability [36], and cardiorespiratory fitness was associated with cognitive flexibility [37]. Similar results were found in studies on normal-weight children and adolescents [38, 39]. Hogan [38] found that children with higher physical fitness levels perform better on cognitive control tasks than children with lower physical fitness levels, especially on tasks of regulating attention demands, and believed there is a positive correlation between health level and executive function performance. Stroth [40] specifically highlighted physical fitness’s effect on executive control.

Therefore, it is possible that physical exercise not only directly affects children’s physical function and executive function, but also affects executive function mediated by physical function.

Nevertheless, there are inconsistent results with the previous studies. For example, some researchers found that physical exercise’s effect to improve physical fitness is weak [41–43]. The same goes on in the studies of executive function [21, 44–47]. A recent systematic review [48] found that the open and closed skills have a different effect on cognitive function. Two of the three studies found that open skills can better improve children’s executive function than closed skills. Another study found that there is no difference between open and close skills for children.

There are several reasons to explain the divergence of research results. Firstly, different physical exercises have distinguishing stimulations on the body fitness and executive function. Some studies suggested that the improvements in cognitive function through physical exercise may be related to the motor movement characteristics of the activities involved [49–51].

Pom cheerleading, a gymnastic sport, is a closed sports skill in which the practitioners perform in a stable and predictable environment and can plan their actions. It is less controlled by the external environment. Performers have sufficient time to prepare, which means the environment change is predictable. Furthermore, pom cheerleading is interesting and highly doable, in line with the characteristics of physiological and psychological development for preschool children [52]. Some studies found that pom cheerleading can significantly improve children’s agility and balance ability [53], and promote their gross motor function and cognitive development [54]. Similarly, some studies found that pom cheerleading can enhance children’s social skills and self-confidence [55]. Moreover, pom cheerleading requires high cognitive participation for preschool children [54, 55]. It will have a significant impact on children’s physical and mental development. Therefore, this study will select pom cheerleading training for children.

Secondly, the characteristics of individual cognitive development were ignored in previous studies. The physical function and cognition of younger children (such as preschool children) are in the stage of rapid development. Compared with older children (such as senior pupils), the similar physical exercise may have a stronger intervention on physical quality and cognition for the younger children. The age of 3–6 is a critical period to develop the executive function, and it is also the peak period for individual brain development [56]. During this stage, the development of executive function is highly sensitive to the individuals’ behaviors. Individual differences caused by a differentiated development level of executive function will produce a series of influences in the following areas, such as the preparedness for school admission [57], academic achievement [58], behavioral adjustment [59] and comprehension ability [60]. So, present study will focus on the development of executive function for preschool children aged 4–5.

Finally, most previous studies discussed the influence of physical exercise on children’s physical function or executive function, or the relationship between physical function and executive function. However, the relationship between these three factors has not been clearly discussed.

**Fig. 1:**
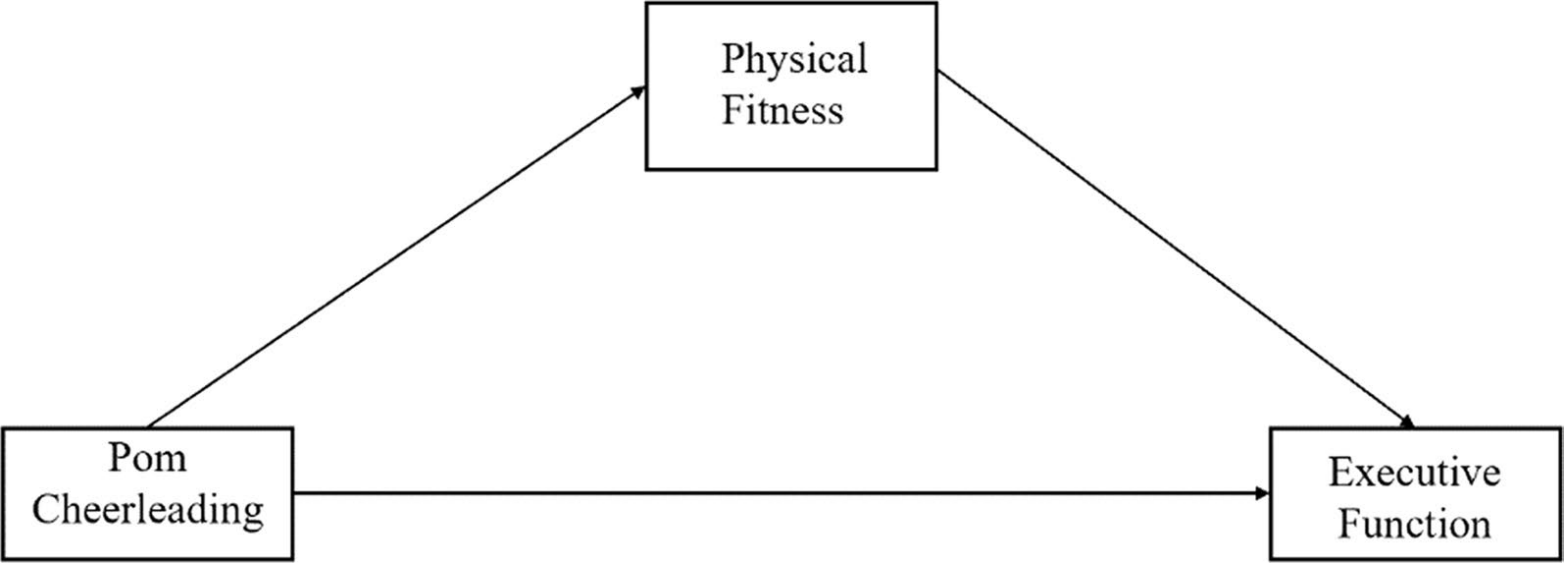
Hypothetical model in this study

Therefore, this study will carry out pom cheerleading training for preschool children to explore the relationship between physical exercise, physical fitness and executive function, and proposes Hypothesis 1: pom cheerleading could improve the physical fitness of preschool children; Hypothesis 2: pom cheerleading could improve the executive function of preschool children; Hypothesis 3: pom cheerleading could improve the executive function of preschool children mediated by physical fitness. The hypothesis model is shown in Fig. 1.

## Experimental methods

### Selection and arrangement of experimental subjects

The G * power 3.1.9 software was used to estimate the sample size. Effect size = 0.25, α error = 0.05. It was calculated that the statistical power of 0.95 can be reached with 54 subjects.

With the prior consent of parents and teachers, a total of 71 children aged 4–5 in the kindergarten affiliated to Henan Normal University were selected as the experiment participants. They are all healthy and without brain trauma or other diseases that may affect physical function or cognition. Children are randomly divided into the experimental group and control group. Children in the experimental group received 40 min of pom cheerleading intervention training four times a week for a total of 12 weeks, while the children in control group carried out routine exercises at the same time. Physical fitness and executive function tests are performed on both groups one week before and after the experiment respectively. Eleven children (six in experimental group and 5 in control group) were excluded from data analysis because they could not complete the final tests. The experiment design was approved by the Review Board of Henan Normal University. The ethics approval code is HNSD-JYXB-2221BS0502. All methods were performed in accordance with the Declaration of Helsinki.

### Measurements of physical fitness

China’s National Physical Fitness Standard Manual (Children) [54, 65] were used to test participants’ lower limb strength (standing long jump, cm), upper limb strength (throwing tennis, cm), speed (10-m return running, s), agility (two-feet continuous jumping, s), balance (walking the balance beam, s), and flexibility (sitting trunk flexion, cm).

### Measurements of executive function

Considering the cognitive development level of preschool children, simple and complex tasks are mixed to measure the three sub-functions of executive function referring to previous similar studies [45, 61, 62]. All participants complete the three tasks in sequence individually in the kindergarten.

Inhibitory control task: The Panda/Lion test [63] is used as the simple inhibitory control test, with 10 trials in total. Test procedure: researcher takes out the panda or lion toys and tells the children “The panda is a good boy, and you should listen to him. The lion is a bad guy and don’t listen to him.” For example, researcher takes out the panda toy and tells children to touch their noses, and children should touch their noses accordingly. Scoring criteria: 3 points for completely correct action, 2 points for partially correct action, 1 point for wrong action and 0 point for no action. The Grass-Snow task [64] was used as the complex inhibitory control test, with a total of 10 trials and a total score of 10 points. Test procedure: First to ensure that children can recognize the color of green grass and white snow. When researcher says “white snow”, children point to the green card. When researcher says “green grass”, children point to the white card. One point for correct action and zero point for wrong action.

Working memory task: The Corsi Blocks Test [65] was used as simple task, with 15 trials and a total score of 15 points. The test procedure is as follows: researcher randomly puts 9 building blocks on a board, and knocks a certain number of blocks each time (starting from 2 blocks and increasing to 6 blocks in turn). The children were required to repeat what researcher did in sequence. One point for correct action and zero point for wrong action. Complex working memory was tested with Reverse Coris Blocks Test, with 15 trials and a total score of 15 points. The only difference from the simple task is that children are required to knock the building blocks in reverse order.

Cognitive flexibility task: The Flexible Item Selection Task (FIST) [66] was adopted, with 18 trials and a total score of 18 points. This task mainly examines children’s ability to flexibly use rules and switch between different dimensions. In each trial, the research shows children a picture with three figures (such as a blue boat, a blue rabbit, and a red rabbit). First, the children are required to point out the two matching figures according to a particular dimension (such as shape: a blue rabbit and a red rabbit), and then let the children point out the two matching figures according to a particular dimension (such as color: a blue rabbit and a blue boat).

### Statistical analysis

(1) SPSS 22.0 analysis software was used to carry out repeated measurement analysis of variance for our 2 (pre-test and post-test) * 2 (experimental group and control group) experimental design, in which physical fitness and executive function as dependent variables; (2) Mplus 7.0 analysis software was used to construct an intermediary model among pom cheerleading, physical fitness and executive function.

## Results

### Homogeneity test of experimental subjects

The independent samples T-test was carried out for participants’ physical fitness and executive function between the two groups before intervention. The results (see Table 1) found that the two groups remain at a similar level of physical and executive function, without distinctive differences.

**Table 1:** The physical fitness and executive function of each group before the intervention (M ± SD)

Variables	Dimensions	Experimental group	Control group	t	P
Physical fitness	Speed (s)	7.47 ± 0.91	7.50 ± 0.84	− 0.12	0.21
	Lower limb strength (cm)	80.13 ± 9.29	79.17 ± 9.14	0.41	0.69
	Flexibility (cm)	10.82 ± 4.01	11.20 ± 3.85	− 0.38	0.80
	Agility (s)	6.28 ± 0.98	6.00 ± 1.01	1.09	0.78
	Upper body strength (cm)	444.3 ± 57.35	442.90 ± 57.41	0.09	0.94
	Balance (s)	6.16 ± 0.99	6.25 ± 0.99	− 0.37	0.10
Executive function	Inhibitory control	27.90 ± 3.21	28.03 ± 4.05	− 0.14	0.11
	Working memory	6.73 ± 3.00	6.63 ± 2.31	0.14	0.09
	Cognitive flexibility	7.03 ± 3.29	7.07 ± 2.85	− 0.04	0.28

### The intervention effect of pom cheerleading on physical fitness

Table 2 shows the results of descriptive statistics for physical fitness of the two groups before and after the intervention. The results of repeated measurement analysis of variance revealed that, in terms of speed, there was a significant interaction between test time and group (F_(1,57)_ = 5.534, p < 0.05, η_p_^2^ = 0.088). The simple effect analysis showed there was a significant difference for experimental group between pre- and post-test (p < 0.01) and no significant difference for the control group. It suggested that systematic pom cheerleading training can improve the speed quality for preschool children.

**Table 2:** Descriptive statistical analysis and results of ANOVA for physical fitness of the two groups before and after the intervention (M ± SD)

Group	Test	Physical fitness					
		Spe. (s)	Lower str. (cm)	Flexi. (cm)	Agil. (s)	Up. str. (cm)	Bal. (s)
Experimental	Pre-test	7.47 ± 0.91	80.13 ± 9.29	10.82 ± 4.01	6.28 ± 0.98	444.3 ± 57.35	6.16 ± 0.99
	Post-test	7.00 ± 0.92	82.03 ± 8.38	11.47 ± 4.07	4.77 ± 0.54	463.73 ± 63.84	5.98 ± 1.03
Control	Pre-test	7.50 ± 0.84	79.17 ± 9.14	11.20 ± 3.85	6.00 ± 1.01	442.90 ± 57.41	6.25 ± 0.99
	Post-test	7.42 ± 0.76	80.57 ± 10.06	11.73 ± 4.08	5.84 ± 1.08	450.53 ± 42.96	6.19 ± 0.80
F_(1,57)_(Interaction)		5.534*	0.273	0.948	45.790**	0.895	0.160
η_p_^2^		0.088	0.005	0.016	0.445	0.015	0.003

Spe. = Speed, Lower str. =  lower limb strength, Flexi. = Flexibility, Agil. = Agility, Up. str. = Upper body strength, Bal. = Balance

*p < 0.05, **p < 0.01

For agility, the interaction between test time and group was significant (F_(1,57)_ = 45.790, p < 0.001, η_p_^2^ = 0.445). The simple effect analysis showed a significant difference between pre- and post-test for the experimental group (p < 0.001), but no significant difference for control group. It revealed that the children’s agility was enhanced after 12 weeks’ pom cheerleading training.

There were no significant interaction between test time and group in terms of lower limb strength (F_(1,57)_ = 0.273, p > 0.05, η_p_^2^ = 0.005), flexibility quality (F_(1,57)_ = 0.948, p > 0.05, η_p_^2^ = 0.016), upper limb strength (F_(1,57)_ = 0.895, p > 0.05, η_p_^2^ = 0.015), and balance ability (F_(1,57)_ = 0.160, p > 0.05, η_p_^2^ = 0.003).

### Effect of pom cheerleading intervention on executive function

Table 3 shows the results of descriptive statistics for executive function of two groups before and after the intervention. The results of repeated measurement analysis of variance showed that the interaction between test time and group was significant in terms of inhibitory control (F_(1,57)_ = 59.289, p < 0.001, η_p_^2^ = 0.510), and the simple effect analysis shows a significant difference for experimental group between pre- and post-test (p < 0.001) and no significant difference for control group.

**Table 3:** Descriptive statistical analysis and results of ANOVA for executive function of the two groups before and after the intervention (M ± SD)

Group	Test	Executive function		
		Inhibitory control	Working memory	Cognitive flexibility
Experimental	Pre-test	27.90 ± 3.21	6.73 ± 3.00	7.03 ± 3.29
	Post-test	34.83 ± 2.55	7.40 ± 2.98	7.37 ± 3.11
Control	Pre-test	28.03 ± 4.05	6.63 ± 2.31	7.07 ± 2.85
	Post-test	29.17 ± 3.40	7.00 ± 2.36	7.23 ± 2.75
F_(1,57)_ (Interaction)		59.289**	19.220*	0.038
η_p_^2^		0.510	0.201	0.006

*p < 0.05, **p < 0.01

Similarly, there was a significant interaction between test time and group in terms of working memory (F_(1,57)_ = 19.220, p < 0.05, η_p_^2^ = 0.201), and the simple effect analysis showed a significant difference for experimental group between pre- and post-test (p < 0.01) and no significant difference for control group.

The above results suggest that 12-week pom cheerleading training can significantly improve preschool children’s inhibitory control and working memory.

In terms of cognitive flexibility, there was no significant interaction between test time and group (F_(1,57)_ = 0.038, p > 0.05, η_p_^2^ = 0.006), which suggested that preschool children’s cognitive flexibility has not been enhanced by the pom cheerleading training.

### The mediating role of physical fitness between pom cheerleading training and executive function

**Fig. 2:**
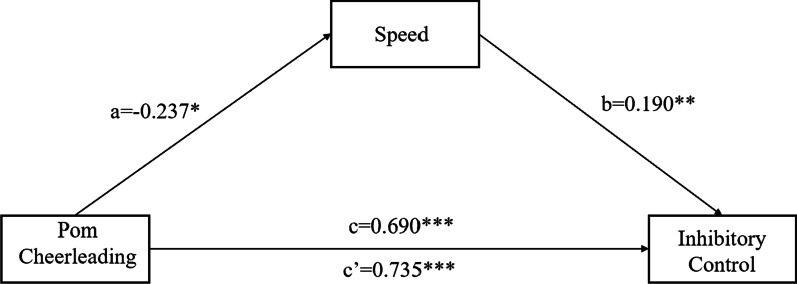
The mediation model of pom cheerleading training, speed, and inhibitory control

Based on the repeated measurement analysis of variance, the group was converted into a dummy variable (0 represents the control group and 1 represents the experimental group) and was included in the structural equation model as an independent variable. Meanwhile, post-test speed quality as a mediation variable and post-test inhibitory control as a dependent variable were included into the model for testing the mediating effect, in which the pre-test speed and inhibitory control were included as control variables (the path of control variable was not presented in the model for simplicity reasons). The coefficients of each path were shown in Fig. 2.

The fit index of the model is as follows: χ^2^/df = 2.835, CFI = 0.973, TLI = 0.920, RMSEA = 0.062. This model has a good fit. It was found that pom cheerleading training can positively predict inhibitory control (β = 0.690, p < 0.001, 95% CI = (0.573, 0.790)). Adding the mediation variable, pom cheerleading training can negatively predict the speed (β = − 0.237, p < 0.05, 95% CI = (− 0.408, − 0.038)). And the speed can positively predict inhibitory control (β = 0.190, p < 0.05, 95% CI = (0.021, 0.406)), while pom cheerleading training can positively predict inhibitory control (β = 0.735, p < 0.001, 95% CI = (0.630, 0.860)). The indirect effect of path (pom cheerleading training → speed → inhibitory control) is 0.052 (95% CI = (− 0.389, − 0.021)), accounting for 5.7% of the total effect. It reveals that speed plays a partial mediating role in the impact of pom cheerleading training on inhibitory control.

**Fig. 3:**
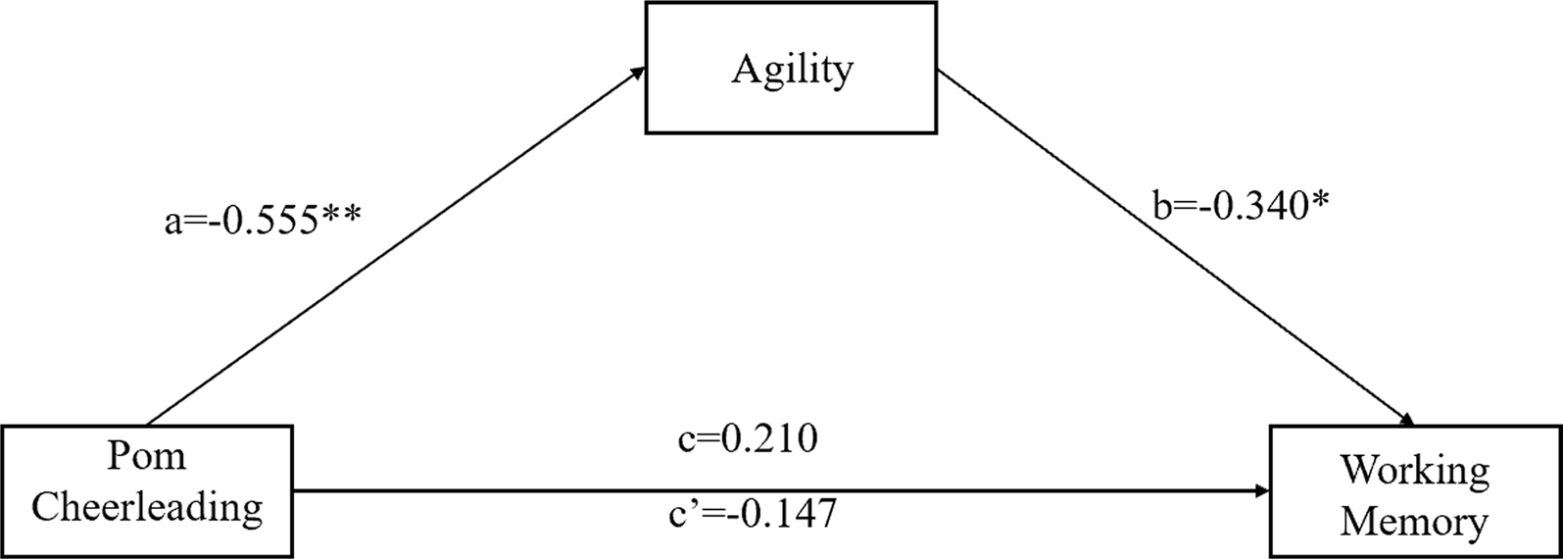
The mediation model of pom cheerleading training, agility and working memory

The group was converted into dummy variable the same way as mentioned before and was included in the structural equation model as an independent variable. Meanwhile, post-test agility as a mediation variable and post-test working memory as a dependent variable were included into the model for testing mediation effect, in which the pre-test agility and working memory were included as control variables. The coefficients of each path are shown in Fig. 3.

The fit index of the model is as follows: χ^2^/df = 3.092, CFI = 0.952, TLI = 0.902, RMSEA = 0.065. This model has a good fit. Pom cheerleading training cannot predict working memory (β = 0.210, p > 0.05, 95% CI = (− 0.174, 0.323)). Adding the mediation variable, the pom cheerleading training can negatively predict agility (β = − 0.555, p < 0.05, 95% CI = (− 0.641, − 0.483)). And agility negatively predict working memory (β = − 0.340, p < 0.05, 95% CI = (− 0.637, − 0.247)), while pom cheerleading training cannot predict working memory (β = -0.147, p > 0.05, 95%CI = (− 0.364, 0.037)). The indirect effect of this path (pom cheerleading training → agility → working memory) is -0.19 (95%, CI = (− 0.472, − 0.087)), accounting for 56.3% of the total effect. It shows that agility plays a major mediating role in the impact of pom cheerleading training on working memory.

## Discussion

In this study, 36 preschool children were intervened with pom cheerleading, and their physical fitness and executive function were compared with the other 35 preschool children who received routine physical trainings. The purpose of current study is twofold. Firstly, it demonstrates the promoting effect of pom cheerleading on preschool children’s physical quality and executive function; Secondly, it constructs mediation models of pom cheerleading intervention, physical quality, and executive function, and reveals the path of how physical exercise influences preschool children’s physical fitness and executive function.

Physical exercise can improve physical health and cognitive function primarily through skeletal muscle energy expenditure [19]. According to the previous research, there are multiple mechanisms to explain the positive effects of physical exercise, such as improvements in angiogenesis, blood oxygen saturation, glucose delivery, cerebral blood flow, neurotransmitter levels [67], brain function [68], and changes in brain volume structure [69].

In this study, we found that the 12-week pom cheerleading significantly improves preschool children’s agility and speed. Children’s flexibility, balance ability, upper and lower limb strength have no distinct changes. These results partially verify the hypothesis 1 of this study. Also, some of these findings are consistent with the research of Zhang [70] which found the 18-week cheerleading can improve agility and limb strength of children aged 9–10 but not increase their cardiopulmonary endurance and speed quality.

Pom cheerleading requires team members to have dancing and pom skills [71]. It has the features like competitiveness, entertainment, artistry, and appreciation. During the performance, performers need to consider various factors such as composition, space, music, intellectual education, aesthetics, time, action and so on. As for the pom cheerleading especially designed for preschool children, it presents different graph formats according to the music rhythm, through changes in running direction and body movements.

Through the systematic pom cheerleading training, children’s body and central nervous system can be frequently stimulated and connected simultaneously, which is reflected in the process of resisting muscle contraction and relaxation frequently, and cooperation with synergetic muscle. As a result, the speed quality is improved, with a gradual enhancement of agility. In terms of behavior, it is more accurate and agile in space and time, and has higher work performance.

There was no significant change in flexibility, balance ability, upper and lower limb strength, probably because there are fewer corresponding stimuli involved in the intervention. Different sports skills have different requirements for physical quality, similarly, and have different effects on physical quality. In addition, according to the physiological development characteristics of preschool children, children have a good flexibility and such flexibility can be further improved only after professional and strict trainings. The strength of the upper and lower limbs belongs to large muscle groups and develops slowly. According to physiological theory, it is not recommended to carry out specialized exercises to improve the strength of young children [72]. Children should not carry out heavy-weight trainings like weightlifting that keep muscles in long state of tension. Such kind of training has adverse effects on young children’s muscle development, and proper dynamic physical activity should be selected to develop children’s strength quality [73]. Therefore, our training programs did not focus on the intervention for preschool children’s strength, which may explain why our findings are inconsistent with those of Zhang [70].

Strength is not the only factor that affects balance ability, others like vestibular organs, audio-visual organs, proprioceptors and other organs, and the above-mentioned organs also have influence on balance ability during the growth of preschool children [74]. Therefore, the improvement of balance ability may take a longer time to achieve for preschool children. The above analysis also enlightens us that when training children’s motor skills, we must carry out scientific guidance according to the characteristics of children’s physical development.

In terms of the executive function, preschool children’s inhibitory control and working memory are improved after the 12-week pom cheerleading training. This result partially verifies the hypothesis 2 of this study. Jiang and Zeng [45] also found that the 8-week football games can promote the development of executive function and improve inhibition control ability in preschool children. Similarly, Wang et al. [75] found that the 16-week Taekwondo training can improve the inhibition control ability and cognitive flexibility in children aged 5–6. However, none of the above studies found an improvement in working memory, which may be related to the attributes, characteristics and exercise intensity of the intervention.

Motor skills refer to the ability to precisely control muscle contractions in accurate time and space. Its physiological essence is the voluntary movement of skeletal muscles under the command of the cerebral cortex [76]. According to the classification of motor skills, pom cheerleading belongs to closed motor skills. Performers can plan their actions in advance and show these actions in a stable and predictable environment. They are commanded by the cerebral cortex when completing physical actions. The implementation of this motor skill requires not only the participation of memory, but also a variety of higher-level cognitive abilities, such as perceptual ability, visuospatial ability, attention, multitasking processing, and planning. So, when children train their motor skills, it can activate the relevant brain areas through body movement and stimulate the excitability of brain cells, which is conducive to maintaining brain sensory and perceptual function, improving memory, and activating the prefrontal cortex responsible for executive function.

Research on the brain has confirmed that physical exercise can improve the function of the prefrontal cortex [18]; in addition, according to the formation law of motor skills, this process reflects the excitement and inhibition of cerebral cortex in time and space, from diffusion to gradual concentration, and then to more concentration and accuracy [75]. Therefore, the formation of motor skills promotes the development of internal inhibition control, working memory and cognitive flexibility. Cognitive flexibility was not been improved significantly in this study, mainly because the three subfunctions of EF developed in different order: inhibitory control and working memory developed earlier, and then cognitive flexibility [77]. Moreover, Schmidt et al. [78] believed that cognitive flexibility (compared with inhibitory control and working memory) is a core part of EF and needs a long-term sports intervention to change. The results of this study are in line with the developmental law of executive function.

As for the relationship between physical exercise, physical fitness, and executive function, it was found that speed quality played a partial mediating role between pom cheerleading training and inhibitory control in this study, which is consistent with our hypothesis 3. Motor speed is when the human body passes through a certain distance in periodic motion [76]. It is the basis for individuals to complete their motor skills and various sports. One of the characteristics of pom cheerleading for children is multi-frequency direction-changing run and rapid motor conversion. Continuous pom cheerleading training changed the coordination relationship between muscle groups and joint amplitude, reduced the resistance caused by fighting muscle group tension. The training brings more accurate cooperation of skeletal muscle and joint and accelerates the conversion speed of excitation and inhibition, which is conducive to the rapid improvement of action speed. The sharp increase in speed further stimulates the development of the nervous system, which is reflected in the rapid and accurate connection of temporary neural connections in the cerebral cortex. In this way it enhances the excitement and inhibition of nervous system in time and space, from the initial diffusion state to a gradual concentration, and then to more concentration and accuracy. Furthermore, the continuous strengthening of these nervous systems leads to higher cognitive functions, such as inhibitory control. Therefore, the improvement of preschool children’s inhibitory control under the stimulation of pom cheerleading training is partly due to their development of speed quality.

Moreover, our study revealed that agility plays a major mediating role between pom cheerleading training and working memory, which also supports hypothesis 3 in present study. In other words, the effect of sports intervention on working memory is achieved indirectly by improving children’s agility. Agility is the ability of an individual to quickly change posture, switch movements and adapt to changes [76]. One of its prominent features is that when the environment suddenly changes, individuals can create new actions to adapt to the new conditions. The physiological basis of agility is mainly related to the structure and function of nerves, receptors, and skeletal muscle. During the training process of pom cheerleading, children quickly change direction and run according to the music rhythm, and construct different queue patterns through body movements. All these complex movements are realized by adjustments and controls, such as the cerebral cortex receiving and analyzing information, giving orders immediately, and then regulating the motor organs to complete the corresponding actions. The brain is required to process multiple tasks and respond at the same time, which are the embodiment of working memory. Therefore, the firmer an individual’s action is, the more accurate and agile the body is in space and time during the sports, and the higher the activation and requirements of working memory are, which will eventually promote the development of working memory.

## Conclusions

The 12-week pom cheerleading training can effectively improve preschool children’s speed, agility, inhibitory control and working memory. The improvement of inhibitory control is partly the result of pom cheerleading training, and the increase of speed causes the other part. Working memory improvement is mainly due to the enhancement of children’s agility brought by pom cheerleading training. In addition, the physical quality and cognition of preschool children do not develop parallelly. The improvement of physical quality can further promote cognitive development.

## Limitation and prospect

First of all, the authors only studied the impact of medium intensity exercise on children’s executive function and physical fitness. Some studies [79, 80] revealed that physical exercise with different intensity can exert different influences on individuals. Therefore, future research could explore the impact path of physical exercise on children’s executive function and physical fitness by manipulating exercise intensity.

Secondly, the development of children’s executive function is affected by multiple factors, such as social interaction, dietary interventions and neurodevelopmental levels [81], which may interact with the intervention effect of physical exercise. In this study the above factors are not considered, which can be modified to avoid interference in future researches.

Thirdly, the sample size in this study is small (the final data analysis includes 60 subjects) because of intervention experiment. For the analysis of mediating effect, the direct effect (c′) will be affected by the sample size. Smaller sample size can lead to a larger standard error, which cause more difficult to obtain significant direct effect. In this case, it is easy to get the result of “full mediation”, similar to the model test result of agility in this study. When collecting enough samples, the previous conclusion of “full mediation” may become “partial mediation”. Therefore, the future research should appropriately increase the sample size to effectively test the mechanism of physical fitness in the process of physical exercise affecting executive function.

## Data Availability

The data that support the findings of this study are available from the corresponding author H. W. (Heng Wang, email: nmgwangh@163.com), upon reasonable request.
